# Institutional Amenities

**Published:** 1898-10-22

**Authors:** 


### The Institutional Workshop.

### INSTITUTIONAL AMENITIES.

By a Member of the London Press.

### I.—COURTESY AND DISCOURTESY.

A submission of my views on institutional amenities to an institutional newspaper may be considered somewhat of a bold step on the part of a member of the lay press. But since the looker-on is acknowledged to see the greater part of the game, so an outsider may detect some of the imperfections of an institutional system. It seems somewhat more courteous, too, to lay the case before the institutionalists themselves rather than to make a sensational popular article from it at this season when sensational articles are much in demand. The first question I should like to ask—from the standpoint of an outsider—is this: To which hospital official does the function belong of interviewing strangers, members of the press, and any others who may happen to call with perfectly legitimate and proper inquiries regarding the internal economy and scope of work of the institution? I should mention that I frequently act as the representative of three London dailies, and, whilst prosecuting inquiries as to metropolitan institutions, I have found the procedure varies so widely that I am led to the conclusion that there is no rule or system with regard to the reception at hospitals and institutions of the inquiring stranger. Sometimes on sending in my card bearing the credentials of three very important newspapers—and I beg to remark in parenthesis that my papers are not of the sensational order, and have never been guilty of startling headlines as to institutional "scandals" and reported "mismanagements"—I am referred to the matron, occasionally to the secretary, more often to an under-clerk, whilst in hospitals I could name the only forthcoming official has been the hospital porter. On the other hand, I must in justice cite another which is one of the most popular hospitals in London, and one which never seems to fail in getting needed subscriptions and means to add fresh wings and improvements. At this fortunate institution the busy secretary is never too busy to see both members of the press and other visitors, and is ever ready to answer any inquiries that are necessary on behalf of the newspaper or any other world. At this same hospital, if the secretary be absent or engaged, the chairman or some other official seems to be always on the spot to do the honours in courteous fashion to the stranger within the hospital gates. And I cannot help referring the wide popularity of this hospital very largely to the courtesy and good management which ensures such pleasant reception to the casual visitor.

To make my criticism more valuable to those most nearly involved, I propose to set forth plainly, and without journalistic or literary embellishment, some of the untoward experiences which have fallen to my lot whilst doing a round of the London hospitals. It would serve no public service to mention the names of the particular hospitals in point. I have no desire to bring odium on any institution. My motive in dealing with this subject is to call attention to an existing system which appears to me to militate against the popularity of our hospitals.

I have little doubt that the majority of the institutional workers before whom the experiences of myself and many other press writers are brought will agree that the methods of receiving strangers which appear to be in vogue in London institutions are neither to be commended nor to be imitated. After saying my say as to some of the institutional amenities which have fallen to my share, I will, in all humility as a lay person before an expert audience, make a few suggestions as to the betterment of facilities for affording information to the *bonâ fide* hospital visitor.

I have not yet forgotten a tour of the London hospitals taken with the simple desire to write some "special" articles in three London dailies as to the Christmas necessities of our various metropolitan institutions. I was animated by a sincere desire to do everything in my power to bring money and gifts in kind to the most needy hospitals, so that both the patients and the banking accounts thereof should reap a harvest of good Christmas contributions; and I made a conscientious point of including some of the institutions which were specially poor, hoping to so put their needs before the public that the benevolent might take pity on their empty Christmas coffers. At one of the most impecunious of such institutions I sent in my card with the names of my three "dailies." I was invited to wait in the doorway. Presently the porter brought me back my card and said, "Matron's out. You'd better call again." Now I am a very busy person, and "calling again" at a rush Christmas season is not an easy undertaking. So I asked the porter to take my card to the secretary. I was shown into his office. I briefly explained what I wanted, for I had no doubt he was as busy as I was. All he said was, "I never give information to the press. The matron may be in in an hour or two if you call again." I explained this to be impossible, and pointed out that it would be a pity if his hospital could not be mentioned at Christmas in three London morning papers. He said, "I assure you I don't in the least mind," which, apart from other considerations, was a very unbusinesslike sentiment. So I pursued my quest with a conviction that here was certainly some cause and effect—a hospital terribly crippled for funds showing so little amenity towards those who desired to help and were in a position to do so.

As a tonic to my nerves I went to the French Hospital, where the charming courtesy of everybody, from the hall porter to the most important official, acted as a balm to my feelings, ruffled as these were by a want of ethical politeness at the first institution.

I went then to another hospital, which is notoriously poor, hoping to give a helping hand. Here there was plenty of courtesy, but no information. Unfortunately, both matron and secretary were out. Would I call again an hour later? This I consented to do. Still the matron and secretary were out. One of the sisters saw me, and was pleasant and kind; but she said she could not tell me about the Christmas trees, festivities, and necessities, since she had no official authority to do so. Would I call again? Having made two visits to the hospital, I was unable to spare the time. I left my card and asked that a few lines should be sent, telling me of any special needs, &c. This the sister readily assented to.

But that letter never arrived.

I wended my way further, and at the next hospital was kept waiting just one and a quarter hours in a cold ante-room. At the end of this time I received every information and many apologies for delay. At the same time no apology can cover such a multitude of lost time. And the fact that so little system prevails, that all the important officials of a hospital are absent at the same time, is not calculated to impress the general public with a sense of a high degree of internal efficiency. Again I sought balm in a foreign hospital, where I was at once ushered into the presence of the leading spirit of the institution, who made me feel that I was a most welcome guest, and received as a stranger should be, in genial hospitable fashion. At many hospitals I was kept waiting three-quarters of an hour, sometimes standing in a hall, where the porter might or might not offer me a chair. At other times I was asked into a board-room, usually unwarmed and guiltless of fire.

